# Sex Differences in Response to TNF-Inhibiting Drugs in Patients With Spondyloarthropathies or Inflammatory Bowel Diseases

**DOI:** 10.3389/fphar.2019.00047

**Published:** 2019-01-28

**Authors:** Bruno Laganà, Angelo Zullo, Maria Lia Scribano, Maria Sole Chimenti, Alberto Migliore, Andrea Picchianti Diamanti, Roberto Lorenzetti, Palma Scolieri, Lorenzo Ridola, Elena Ortona, Marina Pierdominici, Vincenzo Bruzzese

**Affiliations:** ^1^Autoimmune Disease Unit, Sant’Andrea Hospital, Sapienza University of Rome, Rome, Italy; ^2^Gastroenterology and Digestive Endoscopy, Nuovo Regina Margherita Hospital, Rome, Italy; ^3^Gastroenterology Unit, San Camillo Forlanini Hospital, Rome, Italy; ^4^Rheumatology Unit, Tor Vergata University of Rome, Rome, Italy; ^5^Rheumatology Unit, San Pietro Fatebenefratelli Hospital, Rome, Italy; ^6^Rheumatology Unit, Nuovo Regina Margherita Hospital, Rome, Italy; ^7^Gastroenterology Unit, Sapienza University of Rome, Polo Pontino, Latina, Italy; ^8^Center for Gender Specific Medicine, Istituto Superiore di Sanità, Rome, Italy

**Keywords:** spondyloarthritis, inflammatory bowel disease, sex differences, adalimumab, infliximab

## Abstract

Spondyloarthritis (SpA) and inflammatory bowel diseases (IBD) are chronic inflammatory diseases characterized by an aberrant immune response and inflammation with a key role for TNF in their pathogenesis. Accordingly, TNF-inhibiting therapy (TNFi) has dramatically improved the management of these diseases. However, about 30% of patients discontinue TNFi for lack of response, loss of response, and side effects and/or adverse events. Thus, the possibility to identify in advance those patients who will have a good response to TNFi would be extremely beneficial. The aim of this study was to investigate differences between males and females with either SpA or IBD in response to TNFi molecules, i.e., infliximab (IFX) and adalimumab (ADA), considering the reasons for TNFi withdraw. Data of 594 patients, 349 with IBD (M/F: 194/155) and 245 with SpA (M/F: 123/122), previously unexposed to TNFi, were collected. In the IBD group, the rate of female patients discontinuing ADA was significantly higher than that of male patients (*p* = 0.03). No difference emerged according to the distribution of reason for discontinuation. Otherwise, a similar discontinuation rate between female and male patients receiving IFX therapy was observed. In the SpA group, the overall discontinuation rate was not different between males and females both for ADA and IFX. However, in patients treated with ADA, males interrupted therapy more frequently than females due to lack of response (*p* = 0.03). In conclusion, the assessment of sex differences in TNFi response could help physicians personalize the therapeutic approach in a sex-oriented perspective.

## Introduction

Chronic inflammatory diseases are a group of pathologies with different clinical features, but with shared pathogenetic mechanisms leading to a not resolving inflammation. SpA refers to a group of inflammatory diseases that cause arthritis, consisting of predominantly axial or peripheral clinical manifestations (Proft and Poddubnyy, 2018). IBD is a group of chronic systemic diseases causing inflammation of the gastrointestinal tract. Ulcerative colitis affects only the large bowel (Ungaro et al., 2017) and Crohn’s disease can involve the whole gastrointestinal tract (Torres et al., 2017). Several lines of evidence support a key pathogenic role for TNF in perpetuating chronic inflammation of SpA and IBD. In SpA, increased TNFα in synovial tissues and sacroiliac joints, associated with bony destruction, osteoproliferation, and synovitis, were found (Chou, 2013; Dubash et al., 2018). In IBD, elevated expression of TNF was detected in involved colonic tissue (Dionne et al., 1997; Matsuda et al., 2009). Moreover, high levels of TNF in serum and in the intestinal lamina propria were found to correlate with disease activity (Murch et al., 1991, 1993; Maeda et al., 1992). The pathogenic effect of TNF is related to its ability to disrupt the integrity of intestinal epithelium and affect the activity of regulatory T cells and regulatory macrophages (Ślebioda and Kmieć, 2014). In the treatment of SpA and IBD, corticosteroids and immunosuppressive agents are used to induce and maintain long-term remission. When these drugs fail to achieve sufficient disease control, biologic agents are used. Indeed, the introduction of TNFi has dramatically improved the management of SpA and IBD. However, about 30% of patients do not respond to TNFi at all or lose their initial response over time. In the time frame from initiation of therapy until response can be judged, non-responding patients suffer from uncontrolled disease. In addition, some patients have to discontinue TNFi because of severe side effects and/or adverse events (Kalden and Schulze-Koops, 2017; Lopetuso et al., 2017). Thus, the ability to identify in advance those patients who will have a good response to TNFi would be extremely beneficial.

Gender medicine deals with studying how diseases differ between men and women in terms of prevention, clinical signs, prognosis, and therapeutic approach (Baggio et al., 2013). In particular, there are sex-related differences in pharmacokinetics and pharmacodynamics, with evident consequence in the efficacy and side effects and/or adverse events of various drugs in the two sexes. This evidence should be considered before starting any therapy.

Of note, both SpA and IBD are characterized by the presence of sex differences in their onset, progression and response to therapy (Rusman et al., 2018a,b; Shah et al., 2018). Regarding TNFi, differences between males and females in response to these drugs have recently been suggested, with males responding better than females (Gonzalez-Lama et al., 2008a,b; Olivera et al., 2017; Rusman et al., 2018a,b). However, the relationship between sex and response to TNFi in SpA and IBD is far to be fully elucidated. Hence, aim of this study was to better investigate differences between males and females with either SpA or IBD in response to two TNFi molecules that dominate the biologic management of these diseases, i.e., IFX and ADA, taking into account the most common reasons for TNFi withdraw, including lack of response, loss of response at follow-up, and side effects and/or adverse events.

## Materials and Methods

This was a retrospective study enrolling IBD and SpA patients in 7 centers (4 Rheumatology Units and 3 Gastroenterology Units). In each center, diagnostic criteria recommended by International guidelines were followed (Gossec et al., 2016; Gomollon et al., 2017; Magro et al., 2017; van der Heijde et al., 2017). Only patients receiving TNFi therapy (ADA; IFX) were considered. Clinical records were reviewed and data were anonymously collected in a specific electronic database. For statistical analyses, the Mann-Whitney *U*-test, Chi-square test and the Fisher’s exact test were used. The significance level was fixed at *p* < 0.05. This study was carried out in accordance with the recommendations of the Declaration of Helsinki and the ethics committee of Azienda Ospedaliera San Camillo Forlanini approved the study (2006/CE Lazio 1).

## Results

Data of 594 patients, 349 with IBD (M/F: 194/155) and 245 with SpA (M/F: 123/122) were collected.

### Inflammatory Bowel Diseases

Clinical characteristics of IBD patients according to sex are shown in Table 1. There were 155 females (mean age 47 ± 16 years and disease duration 133 ± 110 months) and 194 males (mean age 46 ± 14 years and disease duration 144 ± 104 months) with IBD.

**Table 1:** Clinical characteristics of patients with IBD and SpA according to sex.

Patients	IBD		*P*	SpA		*P*
				
Sex	*F*	*M*		*F*	*M*	
Number, n	155	194		122	123	
Age, years (mean ± SD)	47 ± 16	46 ± 14	0.59	53 ± 14	55 ± 13	0.39
Disease duration, months (mean ± SD)	133 ± 110	144 ± 104	0.16	52 ± 71	64 ± 85	0.88
**Adalimumab**, n (%)	77/155 (50%)	85/194 (44%)	0.28	93/122 (76.3%)	76/123 (61.8%)	**0.02**
Treatment discontinuation, n (%)	17/77 (22%)	8/85 (9%)	**0.03**	26/93 (28%)	16/76 (21%)	0.37
Lack of efficacy, n (%)	6/17 (35.3%)	2/8 (25%)	1.0	7/26 (27%)	10/16 (62.5%)	**0.03**
Loss of efficacy, n (%)	7/17 (41.2%)	3/8 (37.5%)	1.0	14/26 (53.8%)	6/16 (37.5%)	0.35
Side/adverse effects, n (%)	4/17 (23.5%)	3/8 (37.5%)	0.6	5/26 (19.2%)	0/16 (0%)	0.14
**Infliximab**, n (%)	78/155 (50%)	109/194 (56%)	0.28	29/122 (23.7%)	47/123 (38.2%)	**0.02**
Infliximab discontinuation, n (%)	25/78 (32%)	30/109 (27.5%)	0.52	18/29 (62%)	22/47 (47%)	0.24
Lack of efficacy, n (%)	8/25 (32%)	3/30 (10%)	0.09	6/18 (33.3%)	8/22 (36.4%)	1.0
Loss of efficacy, n (%)	9/25 (36%)	16/30 (53.3%)	0.28	4/18 (22.2%)	4/22 (18.2%)	1.0
Side/adverse effects, n (%)	8/25(32%)	11/30 (36.7%)	0.78	8/18 (44.5%)	10/22 (45.4%)	1.0


*Bolded values refer to significant values (P < 0.05).*

Overall, 80 (22.9%; 95% CI = 18.8–27.6) patients discontinued therapy for any reason. Among the female patients, 77 (50%) were treated with ADA and 78 (50%) with IFX. The rate (17/77, 22%) of female patients discontinuing ADA was not significantly different to that discontinuing IFX (25/78, 32%). Among the 194 male patients, 85 (44%) patients were treated with ADA and 109 (56%) with IFX. Of note, the rate (30/109, 27.5%) of male patients discontinuing IFX was significantly (*p* = 0.0018) higher than that discontinuing ADA (8/85, 9%). This observation suggested a better success of ADA than IFX treatment in male patients with IBD.

When comparing data according to sex (Table 1), the overall rate of female patients discontinuing ADA (17/77, 22%) was significantly (*p* = 0.03) higher than that of male patients (8/85, 9%). However, no difference emerged according to the distribution of reason for discontinuation, i.e., lack of response, loss of response at follow-up, and side effects and/or adverse events. No significant differences between female and male patients were detected for IFX discontinuation.

**FIGURE 1:**
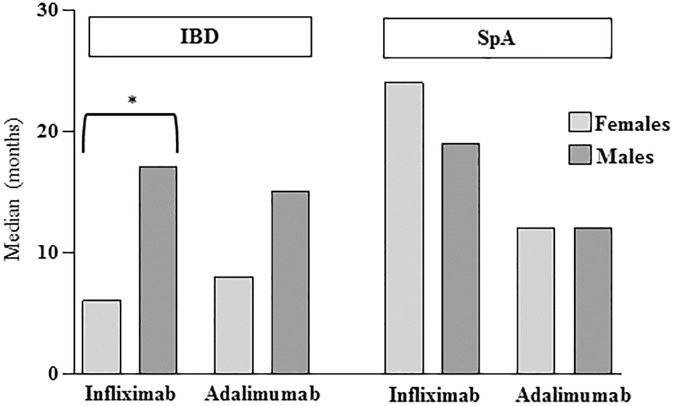
Median of therapy duration before interruption in either Inflammatory Bowel Disease (IBD) or Spondyloarthritis (SpA) patients divided according to sex and type of drug. ^∗^*p* = 0.003 by Mann-Whitney *U*-test.

Analyzing treatment duration before drug discontinuation (Figure 1), we observed that this parameter was significantly shorter in female than male patients for IFX (median 6 months, range 1–50 months vs. 17 months, range 1–146 months, *p* = 0.003) but not for ADA (median 8 months, range 2–48 months vs. 15 months, range 2–35 months).

### Spondyloarthritis

Clinical characteristics of SpA patients according to sex are shown in Table 1.

We enrolled 122 females (mean age 53 ± 14 years and disease duration 52 ± 71 months) and 123 males (mean age 55 ± 13 years and disease duration 64 ± 85 months) with SpA.

Overall, 82 (33.5%; 95% CI = 27.9–39.6) patients discontinued therapy for any reason. Among female patients, 93 patients (76.3%) were treated with ADA and 29 (23.7%) with IFX. The rate of female patients discontinuing IFX was significantly higher than that discontinuing ADA (18/29, 62% vs. 26/93, 28%, *p* = 0.002). Among male patients, 76 (61.8%) were treated with ADA and 47 (38.2%) with IFX. The rate of male patients discontinuing IFX was significantly higher than that discontinuing ADA (22/47, 47% vs. 16/76, 21%, *p* = 0.005). This observation suggested a better success of ADA than IFX treatment in both female and male patients with SpA.

Considering sex differences (Table 1), regarding the choice of TNFi a higher percentage of females than males started ADA and a higher percentage of males than females started IFX (*p* = 0.02 in both cases). The overall discontinuation rate was not different between males and females, however in the ADA therapy group male patients interrupted therapy more frequently than females due to lack of response (10/16, 62.5% vs. 7/26, 27%, *p* = 0.03). As shown in Figure 1, the median of therapy duration before interruption was similar between female and male patients, for both IFX (24 months, range 1–66 months vs. 19 months, range 4–150 months) and ADA (12 months, range 3–90 months vs. 12 months, range 3–72 months).

## Discussion

To investigate whether differences exist between males and females as concerned the response to IFX and ADA, we considered patients with different chronic inflammatory diseases, such as IBD and SpA, disaggregating data for sex and type of drug. Overall, in the IBD patients, we observed that female sex appeared to be a negative predictive factor for ADA response, confirming previous reported studies (Zelinkova et al., 2012; Olivera et al., 2017; Tanaka et al., 2018). In some studies (Zelinkova et al., 2012; Tanaka et al., 2018) ADA discontinuation was observed to be associated to a higher risk in female than in male patients for skin reactions, infections, and arthralgia. In contrast, we did not find any difference between males and females regarding adverse effects underlying ADA discontinuation. Considering that women have a stronger innate and acquired immune response than men, thus being more resistant to infections but more susceptible to autoimmune and allergic reactions (Klein and Flanagan, 2016), the controversial data regarding adverse events needs to be further investigated in a larger patient population. Regarding IFX treatment, no significant differences between female and male patients were detected for drug discontinuation. However, accordingly to Olivera et al. (Olivera et al., 2017), we observed that, among patients discontinuing IFX, females stayed on IFX for a significantly shorter period compared to males. Interestingly, carrying out a head to head comparison of the two TNFi investigated, ADA seems to achieve better results than IFX in males, whereas no differences were observed within the female population.

In the SpA patients, ADA represents the mainstay of management independently from sex. Interestingly, more female patients were treated with ADA whereas more male patients were treated with IFX. No differences for ADA or IFX discontinuation were detected between males and females. Regarding the reason for ADA discontinuation, lack of drug efficacy is more frequent in males than in females. Our data are in contrast with those reported by literature showing that treatment efficacy of TNFi in SpA was lower in women compared to men and that switching TNFi treatment was more frequent in female than in male patients (Rusman et al., 2018a,b). In this regard, one important limitation of our retrospective study is the lack of information regarding a range of variables able to impact TNFi response, such as smoking, body mass index, and glucocorticoid use. Hence, a larger prospective study, taking into account all these variables, is strongly needed to better define the role of sex in TNFi response.

## Conclusion

In conclusion, our data found that patients with chronic inflammatory diseases may have different outcomes linked to the type of drug and the disease, as well as to the sex. In this context, the assessment of sex differences in TNFi response could help physicians personalize the therapeutic approach in a sex-oriented perspective in different inflammatory diseases.

## Author Contributions

BL, AZ, MS, MC, AM, APD, RL, PS, and LR contributed to patient enrolment, data collection, and interpretation. VB provided intellectual input throughout the study. MP and EO provided important contribution to the conception of the work as well manuscript writing. All the authors read and approved the final manuscript.

## Conflict of Interest Statement

The authors declare that the research was conducted in the absence of any commercial or financial relationships that could be construed as a potential conflict of interest.

## Abbreviations

ADA: adalimumab
IBD: inflammatory bowel disease
IFX: infliximab
SpA: spondyloarthritis
TNFi: TNF-inhibiting therapy
